# Effects of CRISPR/Cas9 dosage on TICAM1 and RBL gene mutation rate, embryonic development, hatchability and fry survival in channel catfish

**DOI:** 10.1038/s41598-018-34738-4

**Published:** 2018-11-07

**Authors:** Ahmed Elaswad, Karim Khalil, Zhi Ye, Zhanjiang Liu, Shikai Liu, Eric Peatman, Ramjie Odin, Khoi Vo, David Drescher, Kamal Gosh, Guyu Qin, William Bugg, Nathan Backenstose, Rex Dunham

**Affiliations:** 1 School of Fisheries, Aquaculture and Aquatic Sciences, Auburn University, Alabama, 36849 USA; 2 Department of Animal Wealth Development, Faculty of Veterinary Medicine, Suez Canal University, Ismailia, 41522 Egypt; 3 Anatomy and Embryology Department, Faculty of Veterinary Medicine, Cairo University, Giza, 12211 Egypt; 4 College of Arts and Science, Syracuse University, New York, 13244 USA; 5 College of Fisheries, Ocean University of China, Qingdao, Shandong, 266003 China; 6 Mindanao State University, Maguindanao, 9601 Philippines; 7 Department of Agriculture, University of Maryland, College park, Maryland, 20742 USA; 8 Department of Aquaculture and Fisheries, University of Arkansas, Pine Bluff, Arkansas, 71601 USA; 9 Department of Biological Sciences, University of Manitoba, Winnipeg, Manitoba, R3T 2N2 Canada; 10 Department of Biological Sciences, State University of New York at Buffalo, Buffalo, New York, 14228 USA

## Abstract

The current study was conducted to assess the effects of microinjection of different dosages of guide RNA (gRNA)/Cas9 protein on the mutation rate, embryo survival, embryonic development, hatchability and early fry survival in channel catfish, *Ictalurus punctatus*. Guide RNAs targeting two of the channel catfish immune-related genes, toll/interleukin 1 receptor domain-containing adapter molecule (TICAM 1) and rhamnose binding lectin (RBL) genes, were designed and prepared. Three dosages of gRNA/Cas9 protein (low, 2.5 ng gRNA/7.5 ng Cas9, medium, 5 ng gRNA/15 ng Cas9 and high, 7.5 ng gRNA/22.5 ng Cas9) were microinjected into the yolk of one-cell embryos. Mutation rate increased with higher dosages (*p* < 0.05). Higher dosages increased the mutation frequency in individual embryos where biallelic mutations were detected. For both genes, microinjection procedures increased the embryo mortality (*p* < 0.05). Increasing the dosage of gRNA/Cas9 protein increased the embryo mortality and reduced the hatching percent (*p* < 0.05). Embryonic development was delayed when gRNAs targeting RBL gene were injected. Means of fry survival time were similar for different dosages (*p* > 0.05). The current results lay the foundations for designing gene editing experiments in channel catfish and can be used as a guide for other fish species.

## Introduction

The development of CRISPR/Cas9 system has made genome editing experiments more efficient, precise, rapid, and economical. The system consists of a guide RNA which determines the targeted sequence in the genome and a DNA endonuclease enzyme, Cas9^1,2^. Since 2013, many gene editing experiments have been conducted on fish species including gene knockout^3–7^ and gene insertion or knock-in^8–11^. Recently, extensive genomic data for channel catfish (*Ictalurus punctatus*) became available after the complete sequencing of its genome^12^. However, to implement this genomic data in genetic enhancement programs, it would be necessary to perform functional studies for economically important traits such as growth, feed conversion, tolerance to hypoxia, disease resistance and reproduction^13^. One way to study functional genomics is gene editing using the CRISPR/Cas9 system^14,15^.

Knocking out or silencing a gene may affect not only the primary phenotype, but also one or more secondary phenotypes due to the interactions of gene products in different metabolic pathways resulting in pleiotropic effects^16–18^ or epistasis. In medaka (*Oryzias latipes*), successful knockout of myostatin (*MSTN*) gene increased the expression level of myogenic regulatory factors (*MyoD*, *Myf5* and *myogenin*)^19,20^. MSTN-deficient medaka had faster growth, increased body length and weight, however, the immune system was compromised. Although both mutant and wild type fish had the same mortality percent following immersion challenge of F_3_ MSTN-mutant medaka with red spotted grouper nervous necrosis virus (RGNNV), MSTN-deficient fish had lower expression of several interferon-stimulated genes and higher virus copy numbers^20^.

Microinjection of guide RNA (gRNA)/Cas9 protein into catfish embryos may affect the hatching, embryo development and early fry survival. The higher mortality in injected embryos may be due to the microinjection procedure itself, off target mutations induced by the gRNA/Cas9 protein^21^ or pleiotropic effects of target gene knockout. The extent of off-target mutations depends on the specificity of the CRISPR/Cas9, which is determined mainly by the protospacer adjacent motif (PAM) and the guide RNA sequence^22,23^. Off-target sites with 5 nucleotides mismatch with the gRNA sequence can still be targeted at a frequency that is comparable to on-target site^24^. However, off-target mutations may be minimized by better gRNA design^25–27^, optimization of the dosage of gRNA/Cas9 protein^28^ and the use of Cas9 nickase mutant with paired gRNAs, which reduced off-target effects by 50 to 1,500 fold in cell lines^26,29^.

Since the CRISPR/Cas9 technology opened avenues for precise genome editing, it is important to assess the effects of gRNA and Cas9 on the survival and hatchability of microinjected catfish embryos to allow more effective experimental design for gene editing experiments. Studying such effects would allow accurate estimation of the number of embryos and the amount of gRNA/Cas9 protein to be injected to generate enough founder fish for functional genomics studies with minimal off-target mutations. Thus, the objective of the current study was to assess the effects of microinjection of different dosages of gRNA/Cas9 protein on the mutation rate, embryo survival, embryonic development, hatching percent, early fry survival and deformities in channel catfish. Guide RNAs used in this study were designed to target the channel catfish toll/interleukin 1 receptor domain-containing adapter molecule (TICAM 1) gene and rhamnose binding lectin (RBL) gene separately.

## Results

In this study, we evaluated the effects of microinjection of three dosages of CRISPR/Cas9 protein targeting the channel catfish TICAM 1 and RBL genes on the mutation rate, embryo mortality, hatching, time to hatch, early fry survival and congenital anomalies.

### Analysis of mutation rate in TICAM 1 gene

**Figure 1:**
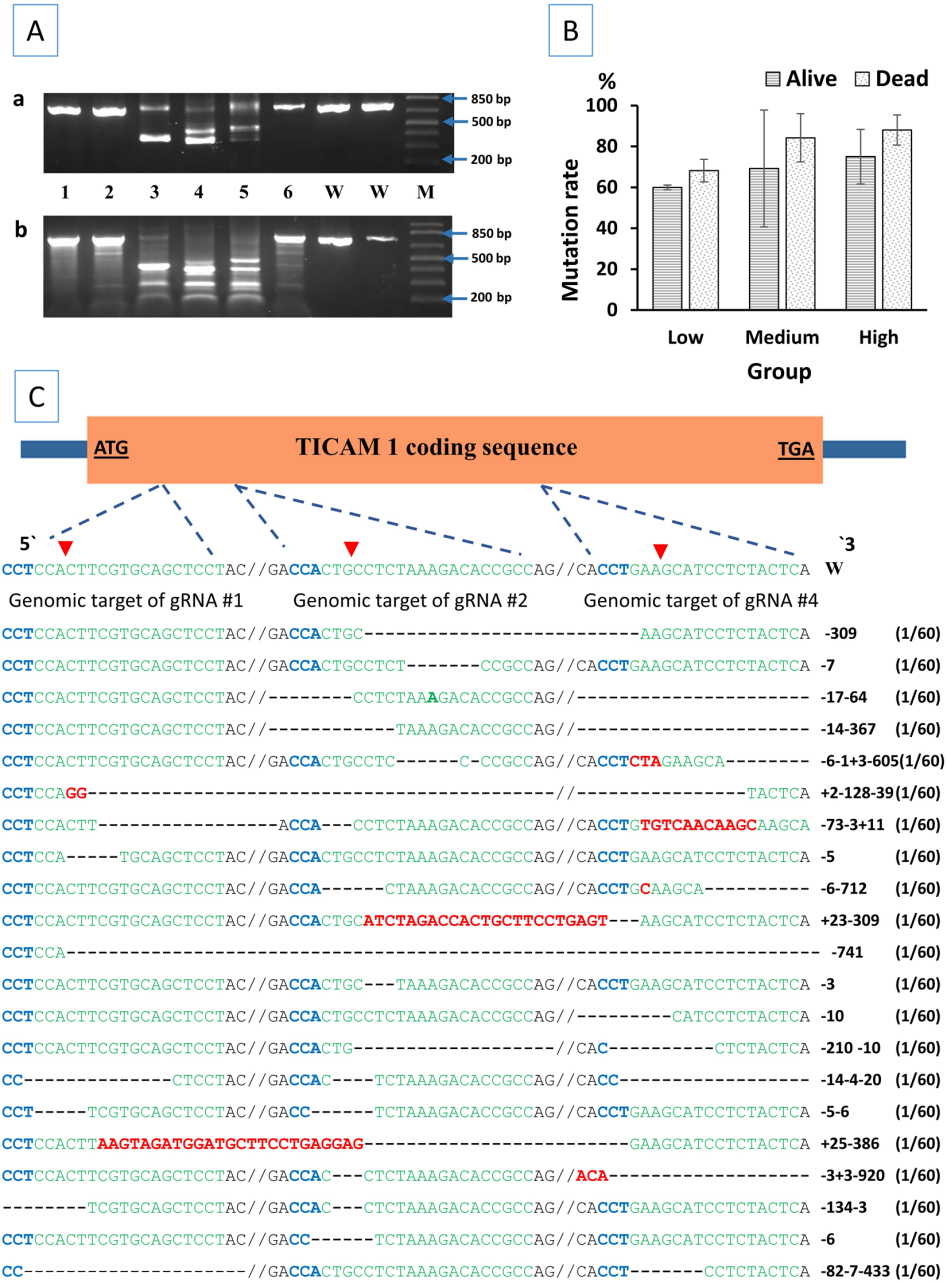
Analysis of mutagenesis efficiencies of CRISPR/Cas9 in the toll/interleukin 1 receptor domain-containing adapter molecule (TICAM1) genomic gene of channel catfish (*Ictalurus punctatus*). (**A**) Identification of mutated TICAM1 gene sequences in channel catfish. For each sample, (a) half of the PCR product was resolved in a 2% agarose gel to detect the large deletion while (b) the other half was digested with Surveyor^®^ enzyme so that (a) and (b) were from the same embryo. Samples 1–6 came from embryos that were microinjected with gRNA/Cas9 protein while lanes W represent wild type channel catfish that were full-sib to embryos 1–6. Embryos with a large deletion had short band(s) when compared to the wild type (750 bp) band. M indicates 1 kb plus DNA ladder (Invitrogen). Electrophoretic results were cropped from the original images shown in Supplementary Fig. S1. (**B**) Mutation rate ± standard error (*SE*) of channel catfish dead embryos and alive fingerlings microinjected with gRNA/Cas9 protein targeting TICAM 1 gene. Three dosages were microinjected into one-cell embryos (low dosage: 2.5 ng gRNA/7.5 ng Cas9, medium dosage: 5 ng gRNA/15 ng Cas9 and high dosage: 7.5 ng gRNA/22.5 ng Cas9). (**C**) CRISPR/Cas9 induced mutations in the TICAM1 gene of channel catfish. The wild-type channel catfish TICAM1 genomic gene structure (which has one exon and no introns) is shown on the top with start and stop codons bold and underlined. Green sequences are the target sites of guide RNAs while the blue sequence (CCT or CCA) represents the PAM (protospacer adjacent motif). Red arrows indicate the expected sites of cleavage by Cas9. Dashes and red letters indicate the deletion/ insertion of nucleotides, respectively. Deletion mutations ranged from few base pair to more than 900 bp deletion. Numbers on the right side of each sequence indicate the number of nucleotides that have been deleted (−) or inserted (+). Double slash is present where a DNA sequence between two gRNA targets has been omitted for simplicity. When double slash is absent, this means that there is a large deletion and the entire sequence between two gRNA targets has been deleted. Mutations are reported in order starting with those at the 5′ end e.g. + 2-128-39 means 2 bp insertion, 128 bp deletion and 39 bp deletion. Each of the mutant alleles was detected once in 60 sequencing reactions (1/60). **1Aa** and **1Ab** were cropped and the full-length gels are presented in Supplementary Figure S1A and S1B, respectively.

A large deletion in TICAM 1 gene was indicated by the shorter band(s) when compared to the wild-type PCR product (750 bp) as shown in Fig. 1Aa. Mutations in some of the samples that did not show shorter bands with PCR were detected with Surveyor^®^ assay. Mutated samples had multiple bands after surveyor^®^ digestion when compared to the surveyor^®^-digested wild-type control (Fig. 1Ab). There was a positive correlation between the gRNA/Cas9 protein dosage and the mutation rate (*r* = 0.445, *n* = 16, *p* = 0.084) (Table 1, Fig. 1B). The mutation rate for the same dosage was higher in dead embryos than 4-month-old fingerlings (Table 1, Fig. 1B). However, the correlation between mutation rate and status (whether individual samples came from dead embryos or 4-month-old fingerlings) was not significant (*r* = 0.337, *n* = 16, *p* = 0.202). DNA sequencing of TICAM 1 pooled samples revealed different types of mutations (Fig. 1C). Deletion mutations ranged from a few base pairs to more than 900 bp deletion. Insertions ranged from a few bp to 25 bp. In one sequencing reactions, a segment of 70 bp was found inverted (TCCTCTACTCAGGAAGTTGGAAAGCATGACAGCTTTAGTACAGAGAAGCAAGCCAGCCAGGAGGAAGAGG). Three of the 4 gRNA (No. 1, 2 and 4) were efficient in inducing mutations. Guide RNA No. 3 was not as efficient as other gRNAs (gRNA No. 3 induced mutation in 3 sequencing reaction out of 60, not listed in Fig. 1C). Six TICAM 1 mutated samples were PCR amplified, pooled into one reaction, cloned into TOPO® vector, transferred to competent cells and sequenced. From this pool of samples, a total of 60 clones were sequenced. Mutations discovered by sequencing are illustrated in Fig. 1C.

**Table 1:** Total number analyzed, number of mutated individuals and mean mutation rate (% of individuals detected with mutations) of channel catfish (*Ictalurus punctatus*) dead embryos and embryos surviving to the fingerling stage (four months) that were microinjected at the one-cell stage with three dosages of gRNA/Cas9 protein targeting toll/interleukin 1 receptor domain-containing adapter molecule (TICAM 1) gene and rhamnose binding lectin (RBL) gene.

Treatment	Dead embryos			Surviving fingerlings		
	Total analyzed	Mutated	Mean mutation % ± *SEM*	Total analyzed	Mutated	Mean mutation % ± *SEM*
TICAM 1*						
Low	22	15	68.2 ± 5.51	50	30	60.0 ± 1.12
Medium	19	16	84.2 ± 11.81	13	9	69.2 ± 28.57
High	25	22	88.0 ± 7.39	16	12	75.0 ± 13.33
RBL**						
Low	26	23	88.5 ± 7.22	33	26	78.8 ± 4.84
Medium	26	24	92.3 ± 5.82	12	10	83.3 ± 2.86
High	28	26	92.9 ± 4.17	14	13	92.9 ± 4.17
TICAM 1 and RBL combined***						
Low	48	38	79.2 ± 5.35	83	56	67.5 ± 4.96
Medium	45	40	88.9 ± 6.17	25	19	76.0 ± 12.18
High	53	48	90.6 ± 4.08	30	25	83.3 ± 7.76

The three gRNA/Cas9 protein dosages were: low dosage: 2.5 ng gRNA/7.5 ng Cas9, medium dosage: 5 ng gRNA/15 ng Cas9 and high dosage: 7.5 ng gRNA/22.5 ng Cas9.

^*^Positive correlation between gRNA/Cas9 dosage and mutation rate (*p* = 0.084).

^**^Positive correlation between gRNA/Cas9 dosage and mutation rate (*p* = 0.025).

^***^Positive correlation between gRNA/Cas9 dosage and mutation rate (*p* = 0.013).

### Analysis of mutation rate in RBL gene

**Figure 2:**
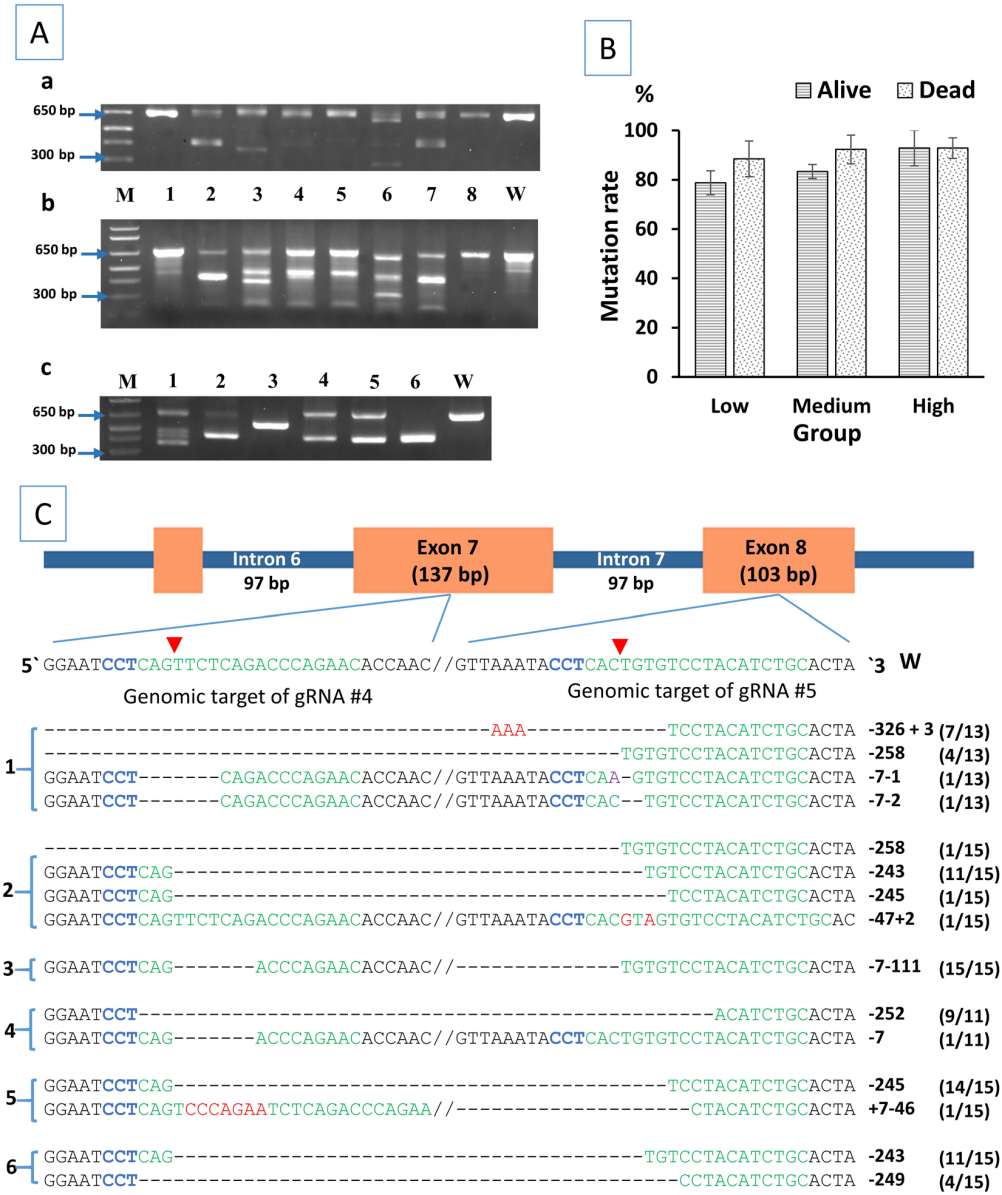
Analysis of mutagenesis efficiencies of CRISPR/ Cas9 in the rhamnose binding lectin (RBL) gene of channel catfish (*Ictalurus punctatus*). (**A**) Identification of mutated RBL gene sequences in channel catfish. One-third of the PCR product from each sample was resolved in a 2% agarose gel to detect the large deletion (a) while two-thirds were digested with Surveyor^®^ enzyme (b) where (a) and (b) came from the same embryo. Samples 1–8 came from embryos microinjected with gRNA/Cas9 protein compared with full-sib wild type control (W). Embryos with a large deletion had shorter band(s) when compared to the wildtype 645 bp band. M indicates 1 kb plus DNA ladder (Invitrogen). (c) PCR products of six microinjected embryo samples that were individually cloned and sequenced where each sample included the DNA from a whole single embryo (sequencing results are illustrated in (C)). (**B**) Mutation rate ± standard error (*SE*) of channel catfish dead embryos and alive fingerlings microinjected at the one-cell stage with three dosages of gRNA/Cas9 protein (low dosage: 2.5 ng gRNA/7.5 ng Cas9, medium dosage: 5 ng gRNA/15 ng Cas9 and high dosage: 7.5 ng gRNA/22.5 ng Cas9). (**C**) CRISPR/Cas9 induced mutations in the RBL gene of channel catfish. Green sequences are the target sites of guide RNAs while the blue sequence (CCT) represents the PAM (protospacer adjacent motif). Red arrows indicate the expected sites of cleavage by Cas9. Samples 1 and 2 were from the low dosage, 3 and 4 from the medium dosage, and 5 and 6 from the high dosage. Dashes and red letters indicate the deletion/insertion of nucleotides, respectively. Numbers on the right side of each sequence indicate the number of nucleotides that have been deleted (−) or inserted (+) e.g. -326-3 means 326 bp deletion and 3 bp insertion. Double slash is present where a DNA sequence between two gRNA targets was omitted for simplicity. Absent double slash means a large deletion where the entire sequence between the two gRNA targets was deleted. Each sequence starting with 5′ and ending with 3′ came from a single reaction representing a single allele. Sequencing reactions with the same type of mutation are represented by a single sequence with a fraction of total e.g. (7/13) means that a certain type of mutation was detected in 7 sequencing reactions out of 13. **2Aa**, **2Ab** and 2Ac were cropped and the full-length gels are presented in Supplementary Figure S1C, S1D and S1E, respectively.

Mutations caused by the injection of RBL gRNAs No. 4 and 5 were detected with PCR (Fig. 2Aa) and surveyor^®^ mutation detection assay (Fig. 2Ab). The two gRNAs induced large deletions detected with PCR as demonstrated in Fig. 2Aa. In some samples, such as sample No. 3 and 6 in Fig. 2Ac, there was no amplification of the wild type band (645 bp) suggesting that Cas9 protein cleaved the target sequence very early in development, possibly at the one cell stage, resulting in biallelic mutations in all cells which was confirmed by DNA sequencing (Fig. 2C). In sample No. 3, a homozygous biallelic mutation was detected. Possible interpretation is that one chromosome was cleaved and repaired by NHEJ then the second chromosome was cleaved and repaired by homologous recombination using the first mutated chromosome as a template resulting in one type of mutation in all cells (Fig. 2C). The six samples presented in Fig. 2Ac came from whole single embryos where each sample represented the DNA from a whole embryo (including all tissues of the same embryo). No abnormal phenotype was observed in the homozygous mutant embryos. Surveyor^®^ assay was used to identify mutant samples that did not have the large deletions as shown in Fig. 2Ab. Samples in Fig. 2Aa and Ab are in the same order. With Surveyor^®^ assay, mutated samples had additional band(s) that were not present in the wild type control (Fig. 2Ab).

Effects of microinjection of different dosages of RBL gRNAs on the mutation rate were similar to TICAM 1 (Table 1). There was a positive correlation between the gRNA/Cas9 protein dosage and the mutation rate (*r* = 0.542, *n* = 17, *p* = 0.025). Except for the high dosage in which dead embryos and 4-month old fingerlings had the same mutation rate, mutation rate for other dosages was higher in dead embryos than 4-month old fingerlings (Table 1, Fig. 2B), but the correlation between mutation rate and status (the individual being from dead embryos or alive fingerlings) was not significant (*r* = 0.263, *n* = 17, *p* = 0.308). DNA sequencing (Fig. 2C) confirmed the presence of mutations which ranged from a few bp to more than 300 bp deletion and a few to several base pair insertions.

### Combined analysis of mutation rate in both TICAM 1 and RBL

The same conclusions were obtained when the mutation data from TICAM 1 and RBL genes were combined (Table 1). Increasing the gRNA/Cas9 protein dosage increased the mutation rate. There was a positive correlation between the gRNA/Cas9 protein dosage and the mutation rate (*r* = 0.429, *n* = 33, *p* = 0.013). Although the mutation rate was higher in dead embryos compared to alive fingerlings, no significant correlation was detected (*r* = 0.245, *n* = 33, *p* = 0.153).

### Observations of congenital anomalies

**Figure 3:**
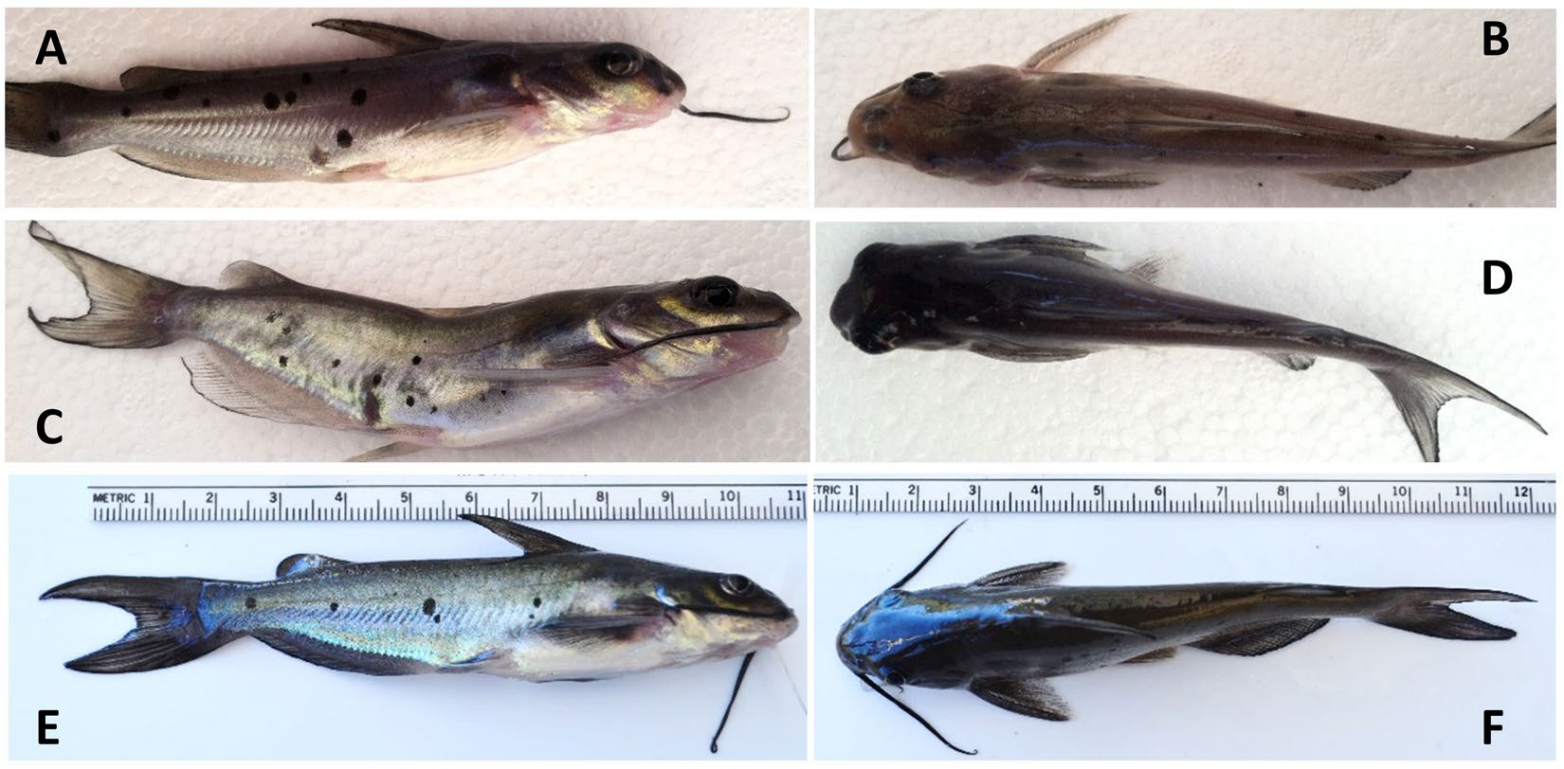
Congenital anomalies in channel catfish (*Ictalurus punctatus*) 4-month-old fingerlings microinjected at the one cell stage with gRNAs/Cas9 protein targeting toll/interleukin 1 receptor domain-containing adapter molecule (TICAM 1) gene and rhamnose binding lectin (RBL) gene. (**A**) Absence of the right barbels. (**B**) Absence of the left eye. (**C**) Spinal deformity. (**D**) The head is curved to the left side with mouth deformity. (**E**,**F**) Normal fish. All injected and non-injected 4-month-old control fry were free of any anomalies. Photos by Ahmed Elaswad.

Congenital anomalies observed in 4-month-old fingerlings included uni- or bilateral absence of eye development, unilateral absence of barbels, spinal and head deformities (Fig. 3A–D). All injected and non-injected 4-month-old control fry were free of any anomalies (Fig. 3E,F). Number, percent and genotype of fish with each type of anomaly are listed in Table 2. Except for one fingerling with head deformity in high dosage treatment of TICAM 1 that was not mutated, all other fingerlings with congenital anomalies had mutated TICAM 1 or RBL genes.

**Table 2:** Number and percent of channel catfish (*Ictalurus punctatus*) 4-month-old fingerlings with congenital anomalies due to microinjection of three different dosages of gRNAs/Cas9 protein targeting toll/interleukin 1 receptor domain-containing adapter molecule (TICAM 1) gene and rhamnose binding lectin (RBL) gene.

Gene	Dosage	Number and percent of fingerlings with anomaly*				Total number
		Absence of eye development	Absence of barbel	Deformed head	Spinal deformity	
TICAM1	Low	0 (0%)	0 (0%)	0 (0%)	0 (0%)	50
	Medium	0 (0%)	0 (0%)	2 (15.4%)	0 (0%)	13
	High	0 (0%)	1 (6.3%)	3 (18.8%) (two were mutated, one was not)	0 (0%)	16
RBL	Low	2 (7.7%)	0 (0%)	1 (3.8%)	1 (3.8%)	26
	Medium	1 (10%)	0 (0%)	0 (0%)	0 (0%)	10
	High	1 (7.7%)	0 (0%)	0 (0%)	0 (0%)	13

Three dosages for each gene were injected including low dosage: 2.5 ng gRNA/7.5 ng Cas9 protein, medium dosage: 5 ng gRNA/15 ng Cas9 protein and high dosage: 7.5 ng gRNA/22.5 ng Cas9 protein. All injected and non-injected 4-month-old control fry were free of any anomalies (see Fig. 3).

^*^All fingerlings with anomalies were mutated except one from the TICAM 1 high dosage that had head deformity but was not mutated.

### Embryo mortality

**Figure 4:**
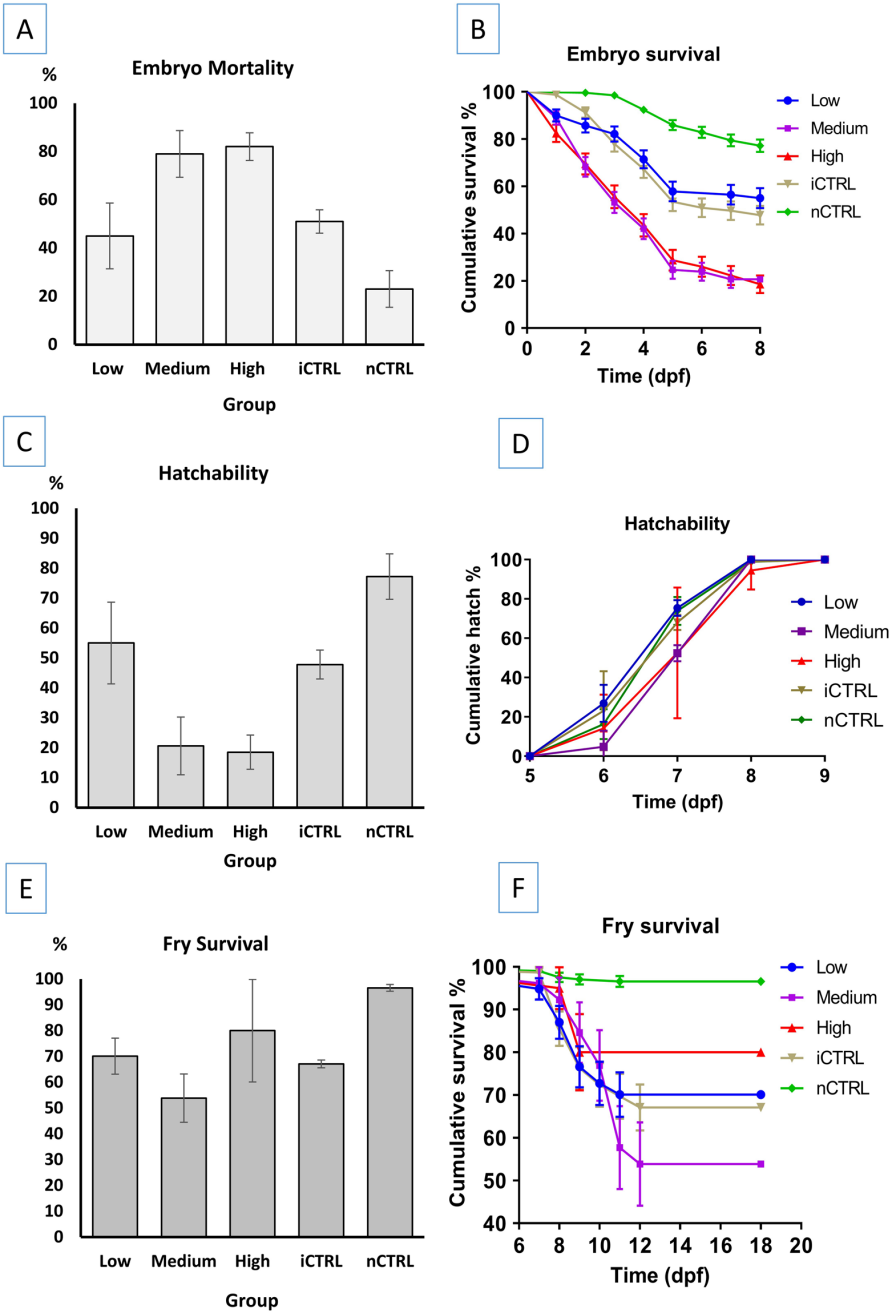
Plots of (**A**) embryo mortality percent, (**B**) mean embryo survival time, (**C**) embryo hatching percent, (**D**) mean time to hatch, (**E**) fry survival percent, and (**F**) mean fry survival time, of channel catfish (*Ictalurus punctatus*) embryos microinjected at the one-cell stage with different dosages of gRNAs/Cas9 protein targeting the toll/interleukin 1 receptor domain-containing adapter molecule (TICAM 1) gene. Two controls were used; injected control embryos (iCTRL) that were full-siblings to the treatment groups and were injected with the same solution and volume but without gRNA or Cas9 protein. The second control was not injected (nCTRL). Guide RNA/Cas9 protein dosages included low dosage: 2.5 ng gRNA/7.5 ng Cas9 protein, medium dosage: 5 ng gRNA/15 ng Cas9 protein and high dosage: 7.5 ng gRNA/22.5 ng Cas9 protein. Hatch rate was calculated as the number of embryos that hatched at a given time-point (day post-fertilization, dpf) compared to the total number of embryos that hatched (see Table 3).

With microinjection of CRISPR/Cas9 protein targeting the channel catfish TICAM 1, embryo mortality started 1 day post fertilization (dpf). Mortality ranged from 23% in the non-injected control (nCTRL) group to 82% in the high dosage group (Table 3, Fig. 4A). Plot of survival curves showed the similarity in mortality between low dosage and the injected control (iCTRL) groups, and medium and high dosage groups (Fig. 4B). The shortest mean time to death for embryos was 4.2 and 4.3 dpf in the medium and high dosage groups, respectively, while the longest was 7.4 days in the nCTRL group (Table 3). Significant differences were detected in embryo mortality in all microinjected groups when compared to the nCTRL group (*p* < 0.0001). Mortality in the low dosage group was not different from the iCTRL group (*p* = 0.295). Mortality in the medium and high dosage groups was not different (*p* = 0.892), however, it was significantly higher in the medium and high groups than all other groups (*p* < 0.0001) (Table 3).

**Table 3:** Total injected embryos, number and percent of dead and alive embryos, mean time to death (days post fertilization, dpf) ± standard error (*SEM*), mean time to hatch (dpf) ± *SEM*, hatch % ± *SEM*, fry survival % ± *SEM* and mean fry survival time (dpf) ± *SEM* for channel catfish (*Ictalurus punctatus*) embryos injected with different dosages of guide RNAs (gRNAs) and Cas9 protein targeting the channel catfish toll/interleukin 1 receptor domain-containing adapter molecule (TICAM 1) gene and rhamnose binding lectin (RBL) gene.

Group	Total injected embryos	Dead embryos		Alive embryos		Embryo mean time to death (dpf) ± *SEM*	Mean time to hatch (dpf) ± *SEM*	Hatch % ± *SEM*	Fry survival % ± *SEM*	Mean fry survival time (dpf) ± *SEM*
		Number	%	Number	%					
**TICAM 1**										
Low	140	63	45.0	77	55.0	6.0 ± 0.22^a^	7.0 ± 0.08^a^	55.0 ± 13.63^ab^	70.1 ± 6.99 ^ab^	15.2 ± 0.49^a^
Medium	126	100	79.4	26	20.6	4.2 ± 0.21^b^	7.4 ± 0.13^a^	20.6 ± 9.65^a^	53.8 ± 9.35 ^ab^	14.3 ± 0.81^a^
High	108	88	81.5	20	18.5	4.3 ± 0.24^b^	7.3 ± 0.18^a^	18.5 ± 5.71^a^	80.0 ± 19.90 ^ab^	16.2 ± 0.83^a^
iCTRL	159	83	52.2	76	47.8	5.9 ± 0.18^a^	7.1 ± 0.10^a^	47.8 ± 4.85^ab^	67.1 ± 1.51 ^a^	15.1 ± 0.49^a^
nCTRL	263	60	22.8	203	77.2	7.4 ± 0.08	7.1 ± 0.05^a^	77.2 ± 7.60^b^	96.6 ± 1.31 ^b^	17.7 ± 0.13
**RBL**										
Low	166	120	72.2	46	27.7	4.6 ± 0.18 ^a^	6.3 ± 0.07^a^	27.7 ± 2.57	80.4 ± 7.06	16.2 ± 0.56^a^
Medium	124	104	83.9	20	16.1	4.2 ± 0.22 ^a^	6.5 ± 0.12^ab^	16.1 ± 0.75 ^a^	75.0 ± 12.37	16.0 ± 0.78^a^
High	173	145	83.8	28	16.2	5.0 ± 0.15 ^a^	6.6 ± 0.10^b^	16.2 ± 1.27 ^a^	62.1 ± 2.75	14.8 ± 0.78^a^
iCTRL	114	34	29.8	80	70.2	5.9 ± 0.17	6.1 ± 0.03^c^	70.2 ± 0.90	97.5 ± 1.27	17.8 ± 0.12^b^
nCTRL	244	32	13.1	212	86.9	6.7 ± 0.07	6.1 ± 0.02^c^	86.9 ± 4.25	93.4 ± 3.39	17.4 ± 0.15^b^
**Combined TICAM1 and RBL**										
Low	306	183	59.8^b^	123	39.8	5.4 ± 0.15	6.7 ± 0.06^b^	40.2 ± 8.68^b^	74.0 ± 4.82^a^	15.6 ± 0.37^ab^
Medium	250	204	81.6^a^	46	18.4	4.3 ± 0.16^a^	7.0 ± 0.11^a^	18.4 ± 4.55^a^	63.0 ± 7.75^a^	15.0 ± 0.58^a^
High	281	232	82.6^a^	49	17.4	4.8 ± 0.14^a^	6.9 ± 0.10 ^ab^	17.4 ± 2.66^a^	69.4 ± 9.25^a^	15.3 ± 0.58^a^
iCTRL	273	117	42.9^b^	156	57.1	6.2 ± 0.14	6.6 ± 0.06^bc^	57.1 ± 5.48^b^	82.7 ± 6.78^a^	16.5 ± 0.27^b^
nCTRL	507	92	18.1	415	81.9	7.5 ± 0.06	6.5 ± 0.04^c^	81.9 ± 4.46	94.9 ± 1.80^a^	17.6 ± 0.10

Three dosages of gRNA were injected including low dosage (2.5 ng gRNAs, 7.5 ng Cas9 protein), medium dosage (5 ng gRNAs, 15 ng Cas9 protein) and high dosage (7.5 ng gRNAs, 22.5 ng Cas9 protein). Two control groups included a control that has been injected with the same volume of injection solution (composition: water with up to one third of 0.5% phenol red solution, no gRNA/Cas9 protein) as the treatment groups which was 50 nanoliters for all microinjected embryos (iCTRL). The second control group was not injected (nCTRL). Significant differences for means in the same column are marked with different superscript letters. Log Rank (Mantel-Cox) test was used for pairwise comparisons of embryo mean time to death, mean time to hatch, and mean fry survival time. One-way ANOVA was used to compare hatch percent and fry survival percent for different groups. When the equality of variance assumption was not satisfied, Welch’s test and Games-Howell test were performed. Significance level used for all tests was *p = *0.05. Means in the same column with the same superscript letters are not significantly different (*p* > 0.05).

**Figure 5:**
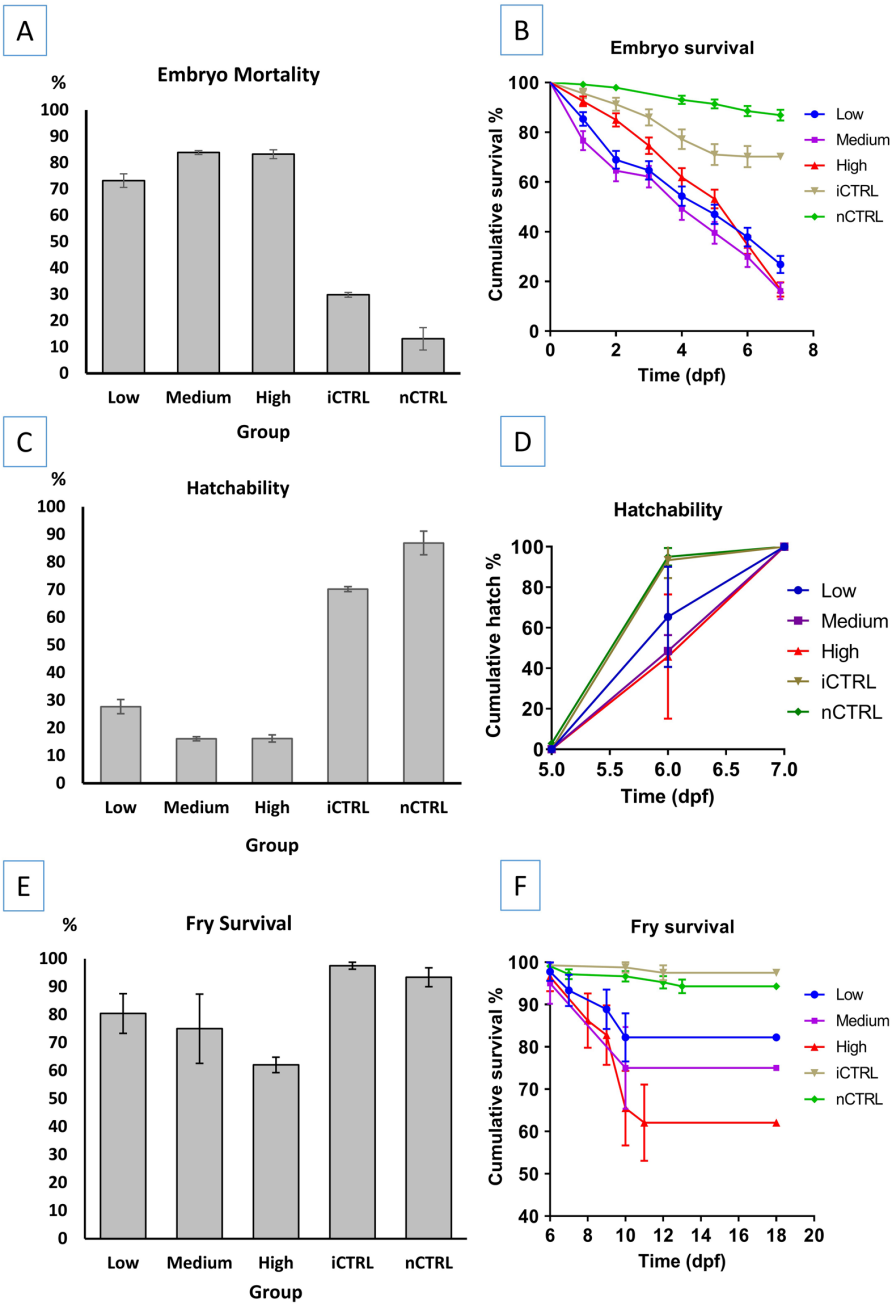
Plots of (**A**) embryo mortality percent, (**B**) mean embryo survival time, (**C**) embryo hatching percent, (**D**) mean time to hatch, (**E**) fry survival percent, and (**F**) mean fry survival time, of channel catfish (*Ictalurus punctatus*) embryos microinjected at the one-cell stage with different dosages of gRNAs/Cas9 protein targeting the rhamnose binding lectin (RBL) gene. Two controls were used; injected control embryos (iCTRL) that were full-siblings to the treatment groups and were injected with the same solution and volumes, but without gRNA or Cas9 protein. The second control was not injected (nCTRL). Guide RNA/Cas9 protein dosages included low dosage: 2.5 ng gRNA/7.5 ng Cas9 protein, medium dosage: 5 ng gRNA/15 ng Cas9 protein and high dosage: 7.5 ng gRNA/22.5 ng Cas9 protein. Hatch rate was calculated as the number of embryos that hatched at a given time-point (day post-fertilization, dpf) compared to the total number of embryos that hatched (see Table 3).

With microinjection of CRISPR/Cas9 protein targeting the channel catfish RBL gene, mortality started at 1 dpf and continued until 7 dpf when all embryos hatched. The lowest mortality percent was recorded in the nCTRL group (13.1%) followed by the iCTRL group (29.8%). The three treatment groups injected with gRNA/Cas9 protein had higher mortality (73.2–83.9%) (Fig. 5A). Significant differences were detected between nCTRL and all three treatment groups (*p* < 0.0001), iCTRL and all three treatment groups (*p* < 0.0001), iCTRL and nCTRL groups (*p* = 0.002) and low and medium dosage groups (*p* = 0.044). Comparison of mortality % between low and high dosages approached the significance level, however, it was not significant (*p* = 0.064). No significant differences in embryo mortality percent were detected between the medium and high dosage groups (*p* = 0.999). The embryo mean time to death ranged from 4.2 to 6.7 days with nCTRL group having the longest time to death (Table 3). Survival curves were plotted (Fig. 5B) and overall comparison of survival curves revealed significant differences in mean time to death between at least two groups (*p* < 0.0001). With pairwise comparisons, the nCTRL group had significantly longer mean time to death than the iCTRL group (*p* < 0.0001) and the two control groups (iCTRL and nCTRL) had significantly longer mean time to death (*p* < 0.0001) than the three treatment groups (low, medium, high dosages). Embryo mean time to death in the medium dosage group was shorter but not significant when compared to the low dosage group (*p* = 0.051) and the high dosage group (*p* = 0.098). Low and high dosage groups had statistically similar embryo mean time to death (*p* = 0.608).

### Embryo hatch

With microinjection of CRISPR/Cas9 protein targeting the channel catfish TICAM1, mean time to hatch ranged from 7.0 to 7.4 dpf for all the groups (Table 3). No significant differences were detected in the time to hatch for all the five groups (*p* = 0.130) (Fig. 4D). Non-injected control had the highest hatch percent (78%) while the high dosage group had the lowest hatch rate (18%) (Fig. 4C). One-way ANOVA revealed significant differences in the hatching percent between at least two groups (*F* = 7.677, *p* = 0.004). Pairwise comparisons detected significant differences in the hatching percent between the medium dosage and nCTRL (*p* = 0.009), high dosage and nCTRL (*p* = 0.005). Hatching percent differences between the low and high dosages approached significance (*p* = 0.09), however, low and high dosages were not significantly different. No differences were detected in all other pairwise comparisons (*p* > 0.05, Table 3).

With microinjection of CRISPR/Cas9 protein targeting the channel catfish RBL, a few individuals in the control groups started to hatch on 5 dpf while most of the fry hatched on 6 dpf. In the treatment groups, fry hatched on 6 and 7 dpf. All fry hatch was completed in all groups on 7 dpf. The highest hatching percent was recorded in the nCTRL group (86.9%) while the lowest hatch percentages were recorded in the medium and high dosage groups (16.1 and 16.2% respectively) (Table 3, Fig. 5C). Comparison of mean hatching percent revealed significant differences between at least two groups (*p* < 0.0001). With pairwise comparisons, the following means for hatching percent were different: low and medium dosage groups (*p* = 0.033), low and high dosage groups (*p* = 0.038), iCTRL and the three treatment groups (*p* < 0.0001), nCTRL and the three treatment groups (*p* < 0.0001) and nCTRL and iCTRL groups (*p* = 0.003). Medium and high dosages had similar means for hatching percent (*p* = 1.000).

Overall comparison of mean time to hatch revealed differences between groups (*p* < 0.0001, Table 3). With pairwise comparisons, the means for time to hatch for the following groups were different: nCTRL and iCTRL hatched earlier than other three treatment groups (*p* < 0.0001). Low dosage group hatched earlier than high dosage group (*p* = 0.039). Low and medium groups had similar mean time to hatch (*p* = 0.184) as well as medium and high groups (*p* = 0.628).

### Early fry survival

With microinjection of CRISPR/Cas9 protein targeting the channel catfish TICAM1, fry started to die one day after hatch. Survival percent ranged from 54 to 97% in medium dosage and nCTRL groups respectively (Fig. 4E, Table 3). Comparison of mean survival percent revealed significant differences between at least two groups (Welch’s test, *p* = 0.001). Games-Howell pairwise comparisons detected significant differences in the survival percent between the iCTRL and nCTRL groups (*p* = 0.001). All other pairwise comparisons were not significant (*p* > 0.05). Accounting for the survival time to death, the nCTRL group had the highest mean survival time to death (17.7 dpf) while the medium dosage group had the shortest mean survival time to death (14.3 dpf) (Table 3). Survival curves were plotted (Fig. 4F) and pairwise comparisons of survival curves revealed significant differences between the nCTRL and iCTRL group (*p* = 0.001), nCTRL and the three treatment groups (low, medium, high dosages) (*p* < 0.0001). All other pairwise comparisons were not significant (*p* > 0.05).

With microinjection of CRISPR/Cas9 protein targeting the channel catfish RBL gene, the mean fry survival ranged from 62.1 to 97.5% with the means of treatment groups lower than the control groups (Table 3; Fig. 5E). The shortest mean survival time to death was recorded in the high dosage group (14.8 dpf) while the longest mean time to death was 17.8 dpf for the iCTRL group. Kaplan-Meier plot of survival curves (Fig. 5F) illustrated the differences in survival time to death between different groups. Overall comparison revealed differences between at least two groups (*p* < 0.0001). Pairwise comparisons detected the significant differences between control and treatment groups in mean time to death. nCTRL and iCTRL groups had similar fry mean time to death (*p* = 0.170). iCTRL group had significantly longer mean time to death when compared to low dosage (*p* = 0.001), medium dosage and high dosage groups (*p* < 0.0001). nCTRL group had significantly longer time to death when compared to the low dosage (*p* = 0.004), medium dosage (*p* = 0.003) and high dosage groups (*p* < 0.0001). Treatment groups had statistically similar mean survival times to death (low and medium *p* = 0.776, low and high *p* = 0.107, medium and high *p* = 0.272).

### Combined analysis of TICAM and RBL

#### Embryo mortality

**Figure 6:**
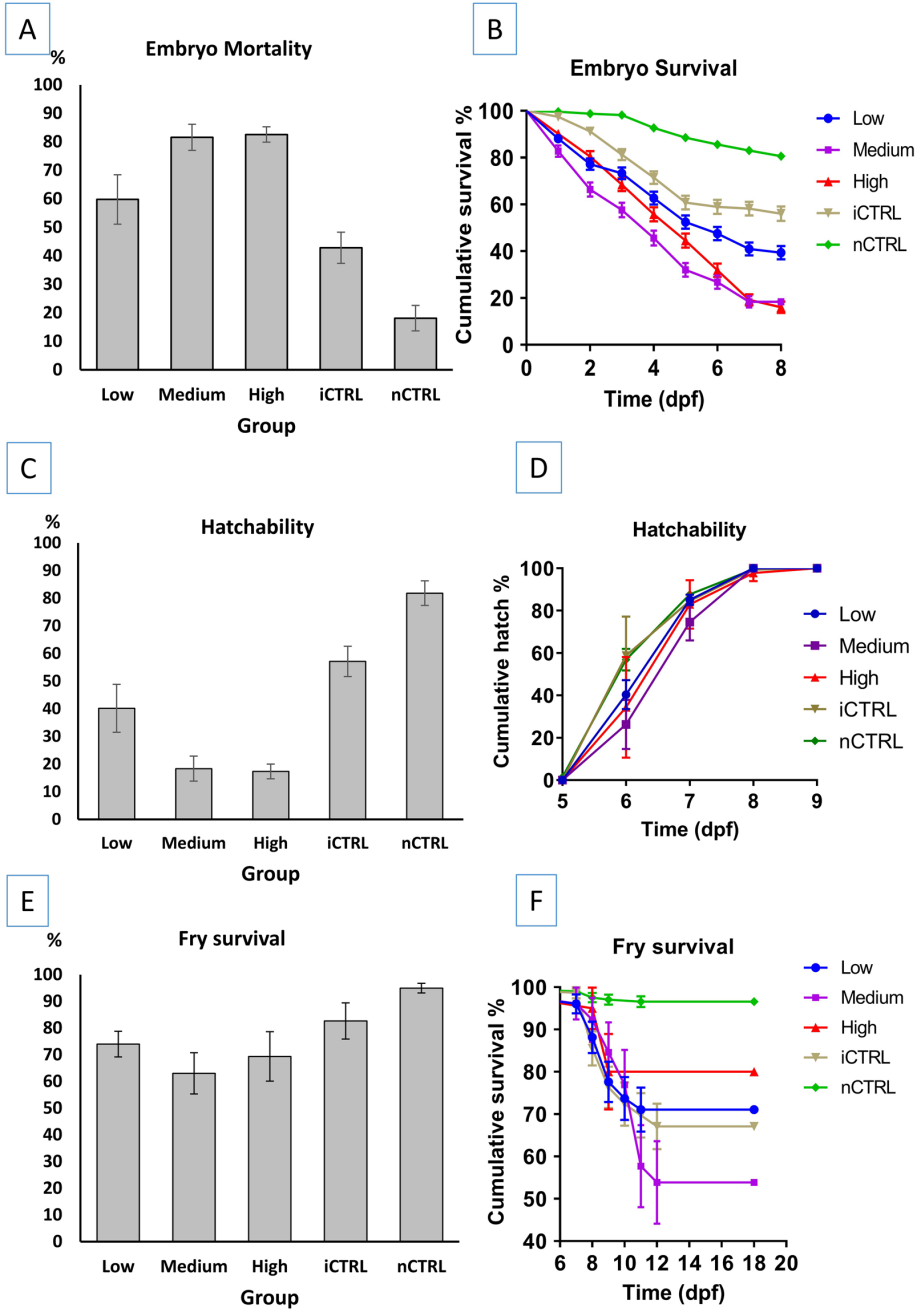
Plots of combined (**A**) embryo mortality percent, (**B**) mean embryo survival time, (**C**) embryo hatching percent, (**D**) mean time to hatch, (**E**) fry survival percent, and (**F**) mean fry survival time, of channel catfish (*Ictalurus punctatus*) embryos microinjected at the one-cell stage with different dosages of sgRNAs/Cas9 protein targeting the toll/interleukin 1 receptor domain-containing adapter molecule (TICAM 1) and rhamnose binding lectin (RBL) genes. Two controls were used for each gene; injected control embryos (iCTRL) were full-siblings to the treatment groups and were injected with the same solution and volumes, but without sgRNA or Cas9 protein. The second control was not injected (nCTRL). Guide RNA/Cas9 protein dosages included low dosage: 2.5 ng gRNA/7.5 ng Cas9 protein, medium dosage: 5 ng gRNA/15 ng Cas9 protein and high dosage: 7.5 ng gRNA/22.5 ng Cas9 protein. Hatch rate was calculated as the number of embryos that hatched at a given time-point (day post-fertilization, dpf) compared to the total number of embryos that hatched (see Table 3).

Embryo mortality percent ranged from 18.1% in the nCTRL group to 82.6% in the high dosage group (Fig. 6A). Overall comparison of mortality % revealed significant differences (*p* = 0.0001) between groups. Significant differences in mortality percent were detected between low and medium dosages (*p* = 0.025), low and high dosages (*p* = 0.006), low dosage and nCTRL (*p* = 0.006), medium dosage and both iCTRL and nCTRL (*p* = 0.004), high dosage and iCTRL (*p* = 0.004), high dosage and nCTRL (*p* = 0.004) and iCTRL and nCTRL (*p* = 0.016). No differences in mortality percent were detected between medium and high dosages (*p* = 0.749) and low dosage and iCTRL group (*p* = 0.150) (Table 3).

Kaplan-Meier test detected significant differences between at least two groups (*p* < 0.0001). Except medium and high dosage which had statistically similar mean time to death (*p* = 0.150), all other pairwise comparisons were different (*p* < 0.0001). nCTRL had the longest mean time to death (7.5 dpf) (*p* < 0.0001), followed by iCTRL which had the second longest mean time to death (6.2 dpf) (*p* < 0.0001). Mean time to death for low dosage group (5.4 dpf) was shorter than nCTRL and iCTRL groups (*p* < 0.0001) and longer than the medium and high dosage groups (*p* < 0.0001) which had a mean survival time of 4.3 and 4.8 dpf respectively (Fig. 6B; Table 3).

#### Embryo hatch

Overall hatching percent was significantly different among the five groups (*p* < 0.0001) (Table 3). Hatching % was statistically different in low and medium dosages (*p* = 0.025), low and high dosages (*p* = 0.006), low and iCTRL (*p* = 0.006), low and nCTRL (*p* = 0.004), medium and iCTRL (0.004), high dosage and iCTRL (*p* = 0.004), high and nCTRL (*p* = 0.004) and iCTRL and nCTRL (*p* = 0.016). No differences in the hatching percent were detected between medium and high dosages (*p* = 0.749) and low and iCTRL (*p* = 0.150) (Fig. 6C; Table 3).

Time to hatch for each group was plotted (Fig. 6D) and comparison of mean time to hatch revealed significant differences between at least two groups (*p* = 0.001). Mean time to hatch was significantly different between nCTRL and low dosage (*p* = 0.022), nCTRL and medium dosage (*p* = 0.0004), nCTRL and high dosage (*p* = 0.016), low and medium dosages (*p* = 0.038) and medium dosage and iCTRL (*p* = 0.005). All other pairwise comparisons of time to hatch were not significant (*p* > 0.05) (Table 3).

#### Fry survival

The highest fry survival percent ± standard error was recorded in the nCTRL group (94.9% ± 4.40) followed by the iCTRL group (82.7% ± 16.62) while the lowest was recorded in the medium group (63.0 ± 18.97) (Fig. 6E), however, fry survival % was not significantly different among all five groups (*p* > 0.05) (Table 3).

Kaplan-Meier test revealed significant differences in the fry mean time to death among groups (*p* < 0.0001). Survival curves were plotted (Fig. 6F) and the fry survival time was longer in the control groups than the treatment groups with the following pairwise comparisons being significant: the three treatment groups (low, medium and high) and nCTRL (*p* < 0.0001), medium and iCTRL (*p* = 0.007), high and iCTRL (*p* = 0.048) and iCTRL and nCTRL (p < 0.0001) (Table 3). All other pairwise comparisons were not significant (*p* > 0.05) (Table 3).

## Discussion

In the current study, the effects of microinjection of three dosages of gRNA/Cas9 protein (low, 2.5 ng gRNA/7.5 ng Cas9, medium, 5 ng gRNA/15 ng Cas9 and high, 7.5 ng gRNA/22.5 ng Cas9) on the mutation rate, embryo survival, hatchability and early fry survival in channel catfish were investigated. Efficient mutagenesis was achieved as demonstrated by PCR, Surveyor^®^ assay and DNA sequencing. The higher dosages achieved higher mutation rates. The mutation rate was 8–15% higher for most dosages in dead embryos compared to surviving 4-month-old fingerlings. Microinjection procedure increased the embryo mortality when injected control embryos were compared to non-injected controls. Injection of gRNA/Cas9 protein increased the embryo mortality and congenital anomalies in 4-month-old fingerlings when compared to the injected control embryos. Hatching percent was reduced from 28% to 16% in RBL and from 55% to about 20% in TICAM 1 when higher dosages of gRNA/Cas9 protein were injected.

Microinjection continues to be the most effective and reliable method to deliver biologically active substances such as DNA, RNA or protein into embryos^30^, however, microinjected embryos had lower survival rates when compared to their non-injected siblings^31,32^. Survival of microinjected embryos depends on the substance and the volume being injected. The larger the volume of microinjection, the higher the embryo mortality^32^. Schubert *et al*.^32^ found that increasing the injection volume increased embryo mortality. In their study, injection of 0.5 nL autoclaved water reduced the survival of zebrafish embryos at 96 h from 98% to 95% while the injection of 4.2 nL reduced the survival to 84% when compared to controls (98%).

Ideal volumes injected into zebrafish (*Danio rerio*) embryos were variable depending on where injection took place. For delivery of injection materials into one cell embryo, 10–20% of the cytoplasmic volume was injected^30^. For microinjection into the yolk, up to 10% of the egg volume can be injected^31^. In case of the fully automated robotic microinjection, 3 nL were injected into single zebrafish embryos^33^ which is 3 times the volume injected by Yin *et al*.^34^. The injection of 3 nL (300 nM) of fluorescein-tagged morpholinos targeting the gene *no tail* into the cytoplasm of one cell zebrafish embryos with an automated microinjection system resulted in 98% survival with 98.5 of surviving embryos exhibiting the intended tailless phenotype^33^. Recently, 2 nL of gene-specific gRNA/Cas9 protein mixture were injected in single embryos for zebrafish embryonic slow muscle gene editing^35^. In this study, 50 nL were injected into the yolk which is about 0.04% of the total egg volume assuming the average diameter of channel catfish eggs is 3 mm^36^.

Part of the embryo mortality in this experiment could be attributed to the microinjection procedures. Comparing the embryo mortality in nCTRL with the iCTRL groups confirmed the adverse effects of microinjection on embryo survival. Embryo survival was reduced from 81.9% to 57.1% due to microinjection of 50 nL of buffer (water and phenol red). The nCTRL group had significantly longer mean time to death and higher survival percent when compared to the iCTRL group. Except for microinjection, embryos in these 2 groups were full-siblings and had been exposed to the same handling stress. The same result was obtained for both genes, TICAM 1 and RBL. A similar conclusion was drawn when zebrafish embryos were microinjected with 0.5 and 4.2 nL of autoclaved water^32^. In their study, non-injected zebrafish embryos had 98% survival at 96 h after vehicle injection, while survival was reduced to 95 and 84% in embryos injected with 0.5 and 4.2 nL of autoclaved water, respectively. In the current study, a coloring material (phenol red) was used to track the injection. Phenol red has been used by several researchers^7,11,34,37–41^, however, the effects of adding phenol red to the injection material on the survival rate of fish embryos have not been investigated, and the increased mortality could be due to the physical damage of the microinjection, the phenol red or both.

Another cause of embryo mortality could be the gRNA/Cas9 protein and its dosage. In the six gRNA/Cas9 treatments for TICAM 1 and RBL genes combined, five treatments (all except the low dose for TICAM1 gene) showed significant effects of gRNA/Cas9 protein where embryo survival was reduced when compared to the iCTRL treatment. For embryo hatching %, the treatment with the lowest hatch rate was the high dosage (17.4% for TICAM 1 and RBL combined) which was still higher than the hatch rate (9%) obtained by Qin^42^ when channel catfish embryos were microinjected in the blastodisc with 100 picograms of gRNA and 300 picograms of Cas9 mRNA targeting gonadotropin-releasing hormone (GnRH) gene. The volume of injection was not reported, and no coloring material was used. Hatch rate was 11% for injected control embryos, which is less than the hatch rate of injected control embryos in the current study (57.1% for TICAM 1 and RBL combined). However, if the ratio of the hatch for the Cas9 injected and the injected controls is examined, the relative hatch of Cas9 mRNA injected embryos was 82% and for the Cas9 protein injected embryos in the current study was 31%. Several factors could be the cause of this difference in the two studies including relative egg quality, differences in the targeted genes and possible off-target mutations, the site of injection and possible differences in the potential pleiotropic and epistatic effects during late embryogenesis, although the immune system is not functional at that time^43^.

Increasing the concentration of TICAM 1 gRNA/Cas9 protein increased the mutation rate of both genes as well as the mortality rate of channel catfish embryos. In zebrafish, a similar conclusion was obtained when varying concentrations of gRNA/Cas9 mRNA and gRNA/Cas9 protein were injected^35,44^. The researchers concluded that increasing the concentration of Cas9 mRNA injected into zebrafish embryos led to increasing occurrence of toxic phenotypes ranging from death after several hours to general problems with the heart, nervous system and axis formation. When higher concentrations of gRNAs targeting the solute carrier family 24 member 5 (SLC24A5) gene were injected into zebrafish embryos, gene knockout, as well as toxicity, increased^44^. This increased mortality in the two studies could be due to off-target effects, due to direct toxicity from increasing concentrations of RNA and protein or both, and the exact cause cannot be ascertained without further study.

DNA sequencing proved the efficiency of CRISPR/Cas9 system for inducing mutations in channel catfish. The frequency of different types of mutations indicates how early in development that CRISPR/Cas9 protein induced-mutations took place. The fewer types of mutations with high frequency indicated cleavage of DNA targets by Cas9 protein at earlier developmental stages. For RBL, low dosage embryos had four types of mutations. In the medium dosage, there were two types of mutations in one mutated embryo while in another one, there was only one type of mutation in all sequencing reactions (15/15) suggesting the possibility of mutating RBL gene at the one-cell stage. No wild type alleles were detected by DNA sequencing in medium and high dosage, providing a strong evidence that both chromosomes were mutated in every cell. The two types of mutation in medium and high dosages suggest bi-allelic mutation of RBL gene in which DSB in each of the two alleles resulted in a different type of mutation. CRISPR/Cas9 induced bi-allelic mutations were reported in zebrafish when the tyrosinase gene was targeted^3^. Tyrosinase mutant embryos were mosaic for pigmentation suggesting that there were still some cells with the wild type tyrosinase gene that produced pigmentation.

TICAM 1 and RBL are immune-related genes that play a significant role in the disease progression pathway of enteric septicemia of catfish and columnaris disease, respectively^45–48^. However, there is no information on their pleiotropic effects and if knockout of the two genes would affect survival. In the current study, successful knockout of those genes has been achieved, so the possibility of the knockout affecting the survival of channel catfish embryos cannot be eliminated without further investigation. Pleiotropic effects for gene knockout were previously reported^16,17^ such as the immune suppression in myostatin-deficient medaka^20^. The possibility of the effects of off-target mutations on embryo survival exists. However, many researchers reported that low off-target frequency is associated with CRISPR/Cas9 system^26,49–51^. In contrast, some researchers reported substantial off-target effects for the CRISPR/Cas9 system^21,24,25^. These conflicting conclusions might be due to the design of gRNA, the method of off-target mutation detection or the genes being targeted^51^. Toxic effects of gRNA depend on the genes that may have been off-targeted, and it is difficult to eliminate this possibility in the current experiment. In RBL, the three dosages of gRNA resulted in statistically similar embryo survival that was different from the iCTRL treatment. However, with more replication, the low dose may result in statistically lower mortality as the embryo survival was 18% higher than the next closest (high) dose for survival.

The introduction of nucleic acids into fish embryos may affect the embryo hatch rate and time^52,53^. In channel catfish, these effects are usually reduced hatch rate and extended hatching time of transgenic embryos when compared to controls^53^. In the current experiment, the effects of different doses of gRNA/Cas9 protein on the mean time to hatch were variable. In TICAM 1, no differences in mean time to hatch were detected between the treatments and controls suggesting that, under the current experimental conditions, neither microinjection procedures nor different doses of gRNA/Cas9 protein had significant effects on hatch time. For RBL, microinjection procedures did not affect the time to hatch (nCTRL and iCTRL groups had similar mean time to hatch). However, gRNA/Cas9 injection delayed the hatch time when compared to controls. Moreover, increasing the dose of gRNA/Cas9 increased the time to hatch (delayed hatch). Su *et al*.^53^ found that the effects of transgenic sterilization constructs introduced by electroporation on embryo hatching time were variable. Some constructs did not affect the mean time to hatch while others delayed the embryo hatch time when compared to controls.

The effects of gRNA/Cas9 on delaying the embryonic development and subsequent extension of embryo time to hatch cannot be eliminated. Those effects may be attributed to the double-strand breaks induced in the DNA. The adverse effects of DNA damage on embryonic development exist and such effects depend on the severity of the DNA damage. In mice, severe sperm DNA damage induced by sperm chromatin fragmentation (SCF) resulted in delayed paternal DNA replication and retarded the embryonic development and a large proportion of the embryos were arrested at the G2/M (second gap phase/mitosis) border^54^. There is also time needed for the cell to repair the DNA double-strand break which may delay embryonic development and result in temporary cell arrest during the cell cycle until the DNA repair is complete^55^. The time needed for repair ranges from several minutes to several hours depending on the type of cells, the repair mechanism and the severity of the DNA double-strand breaks^56–58^.

Microinjection procedure decreased the hatching percent significantly in RBL, while in TICAM 1, the hatch percent in the nCTRL group was still 22% higher than iCTRL group, but the difference was not statistically different due to the high variation in hatch percent between replicates. The injection of gRNA/Cas9 protein reduced the hatch percent significantly in RBL when compared to the iCTRL group, and increasing the dosage from low to medium reduced the hatch percent, which is mainly affected by the embryo mortality in each treatment. Although it was not significant for TICAM 1, the hatch percent was reduced by more than 30% when the dosage was increased from low to medium. Fry in most treatments had significantly lower mean survival time when compared to the nCTRL revealing the possible effects of microinjection and/or the dosage of gRNA/Cas9 on early fry survival. The microinjection procedures significantly reduced the early fry survival in TICAM 1 but gRNA/Cas9 protein did not affect fry survival. In RBL, the gRNA/Cas9 protein reduced fry survival.

The present study indicated that the mutation rate can be increased by increasing the dosage of gRNA/Cas9 protein. Higher dosages achieved both homozygous and heterozygous biallelic mutations whereas no wild type alleles were detected supporting the possibility of gene knockout at the one cell stage in both chromosomes. However, higher dosages reduced embryo survival and hatching. The low dosage resulted in embryos that had both mutated and wild type alleles. Future research is needed to determine the germline transmission rate of mutations and the effects of TICAM 1 and RBL gene knockout on the immune response and disease resistance of channel catfish. Our results lay the foundations for designing gene-editing experiments in catfish to choose the best dosage for tradeoff between the mutation rate and other effects on embryos.

## Materials and Methods

All experimental protocols used in this experiment were approved by the Auburn University Institutional Animal Care and Use Committee (AU-IACUC) before the experiment was initiated, and followed the Association for Assessment and Accreditation of Laboratory Animal Care (AAALAC) protocols and guidelines.

### Experimental design

The experiment included three treatments and two control groups for each of TICAM 1 and RBL genes. Three dosages of gRNA/Cas9 protein for each gene (2.5 ng gRNA/7.5 ng Cas9 protein, 5 ng gRNA/15 ng Cas9 protein and 7.5 ng gRNA/22.5 ng Cas9 protein) were injected (with fixed volume, 50 nL), while the first control group was injected with the same volume of buffer (50 nL) without gRNA or Cas9 protein (injected control, iCTRL). The second control was not microinjected (nCTRL). Each of the treatment and control groups for the 2 genes had three replicates. All embryos in treatment and control groups for each gene were full-siblings, exposed to the same handling stress and reared in the same environmental conditions.

### Preparation of guide RNA and Cas9 protein

Guide RNAs used in the study are listed in Table 4. Four gRNAs for TICAM 1 (GenBank Accession No. DQ423778.1) and 5 gRNAs for RBL (GenBank Accession No. KF725628.1) genes were designed using CRISPRscan gRNA design tool^59^. Guide RNAs were prepared according to Shah *et al*.^44^ using Platinum™ *Taq* DNA Polymerase (Invitrogen)^7^. Cas9 protein was purchased from PNA BIO Inc. (Newbury Park, CA). For TICAM 1 gene, equal amounts of the 4 gRNAs were mixed with Cas9 protein before injection. For RBL, equal amounts of the 5 gRNAs were mixed with Cas9 protein before injection. Each gRNA represented one-fourth and one-fifth of the total gRNAs in the injection solution for TICAM 1 and RBL genes respectively. For each dosage, the concentrations of gRNAs and Cas9 proteins were adjusted so that the injection of 50 nL will deliver approximately the amount needed for each dosage. In all cases, the gRNA/Cas9 protein mixture was incubated on ice for 10 min before use.

**Table 4:** The genomic target sequences for gRNAs used to target the channel catfish (*Ictalurus punctatus*) toll/interleukin 1 receptor domain-containing adapter molecule (TICAM 1) and rhamnose binding lectin (RBL) genes. Underlined sequences represent the protospacer adjacent motif (PAM). (see Figs 1C, 2C).

TICAM 1	RBL
Target sequence for gRNA (3′→5′)	
1. GGAGGTGAAGCACGTCGAGGA	1. GGACTTTGAGTCGGAGAAGTGG
2. GGTGACGGAGATTTCTGTGGCGG	2. GGGGCTGGGTACGAAACCTTGG
3. GGGTTCTCGGGTTTATATCTT	3. GGTGAATGCTGACGTAGTTAC
4. GGTGGACTTCGTAGGAGATGAG	4. GGAGTCAAGAGTCTGGGTCTTG
	5. GGAGTGACACAGGATGTAGACG

### Artificial spawning and microinjection

Kansas random (KR) channel catfish males and females were selected for artificial spawning. Broodstock preparation, artificial spawning, and microinjection procedures were performed according to Elaswad *et al*.^60^. Briefly, female fish were implanted with 85 μg/kg of luteinizing hormone releasing hormone analog (LHRHa) to induce ovulation. Eggs were stripped in a 20-cm greased spawning pan. Males were euthanized, testes collected, crushed, and sperm was prepared in 0.9% saline solution. Eggs were fertilized in batches of 200–300 every 30–60 minutes, and embryo microinjection was initiated 15 min after fertilization and continued for 60–90 min (embryos were still in the one-cell stage).

### Embryo incubation and hatching

Immediately after microinjection, embryos were incubated in Holtfreter’s solution (59 mM NaCl, 0.67 mM KCl, 2.4 mM NaHCO_3_, 0.76 mM CaCl_2_, 1.67 mM MgSO_4_)^61^ with 100 ppm doxycycline^53^. Each replicate of a treatment was incubated in a plastic tub containing 7 liters of Holtfreter’s solution and continuous aeration. Incubation temperature ranged from 26 to 28 °C while dissolved oxygen levels were kept above 5 ppm using air diffusers. All tubs for each gene were held in a rectangular tank containing pond water to reduce the fluctuations in temperatures and ensure all the experimental units had the same temperature.

Dead embryos were removed and recorded daily and assigned a value representing the day of death (dpf). Embryos were not handled in the first 24 hours of incubation. At the time of dead embryo removal, Holtfreter’s solution was replaced and the incubation tubs were cleaned. The temperature of Holtfreter’s solution was monitored and adjusted to the same degree as the old solution to minimize the adverse effects of temperature fluctuation on embryos. The hatching temperature was 28 °C. When hatching began, the time of hatch, the number of hatching fry and the number of dead fry were recorded separately for embryos in each experimental replicate. After hatch, the fry were reared in Holtfreter’s solution without doxycycline until 10 days post fertilization (dpf). When the fry began to swim up (at 9–10 dpf), they were fed *artemia nauplii* (Pentair Aquatic Eco-systems, USA) three times/day to ensure continuous feed supply. Starting at 10 dpf, complete water exchange was done every third day with one-third of Holtfreter’s solution replaced with pond water each time until two-thirds of the Holtfreter’s solution was replaced with pond water. Water quality parameters were monitored daily. The experiment on early fry survival was terminated at 18 dpf when no fry died in the 5 groups for each gene for 5 successive days and the alive fry were reared in 60-L aquaria.

### Mutation detection

Calculation of mutation rates and identification of different types of mutations were investigated at the binding sites for all TICAM 1 gRNAs (No. 1, 2, 3, and 4) and two of five gRNAs for RBL (No. 4 and 5) (Figs 1, 2; Table 4). Genomic DNA was extracted from both whole single embryos that died after 72 h post fertilization and barbel tissue from 3-4-month-old fingerlings. Yolk and egg shells were removed from the embryos before DNA was extracted. DNA extraction was performed using proteinase K digestion and ethanol precipitation^62^. PCR and Surveyor® mutation detection assay were performed to identify the mutant embryos and fingerlings. Primers (Table 5) were designed to amplify a segment of TICAM 1 and RBL genes. The distance between the annealing site for the primers and the binding sites for gRNAs was not less than 100 bp from both ends.

**Table 5:** Primers used to amplify a partial sequence of channel catfish (*Ictalurus punctatus*) toll/interleukin 1 receptor domain-containing adapter molecule (TICAM 1) gene and rhamnose binding lectin (RBL) gene flanking the target sites for guide RNAs.

Primer name	Strand	Sequence 5′-3′	Annealing temperature	Product length (bp)
T1F	Forward	GCTGCTGAATGTCTGATTATG	60 °C	750
T1R	Reverse	GTCCTCCACACTCCTGAAG		
T2F	Forward	ACTGGTGGACGAGAAGAAG	60 °C	1462
T2R	Reverse	CTGGATGTGGATGTTGGATG		
RBL F	Forward	ATCGTGTTGTGATCTGTGAG	57 °C	645
RBL R	Reverse	GCCCTAGCCAATTTGATGTT		

Primers T1F and T1R were used to amplify DNA segments containing small indels while primers T2F and T2R were used for the large deletions (see Fig. 1A). Primers RBL F and RBL R were used to amplify a partial DNA sequence of the RBL gene (see Fig. 2A).

PCR was performed using Expand High Fidelity^PLUS^ PCR System (Roche Diagnostics, Indianapolis, IN, USA) with the following components: up to 20 µl PCR grade water; 1X Expand HiFi^PLUS^ reaction buffer with MgCl_2_, 0.2 mM dNTP mix, 0.4 µM for each of the forward and reverse primer of the same set, 100–300 ng genomic DNA and 1.25 units of Expand HiFi^PLUS^ enzyme blend. PCR cycling conditions were as follows: initial denaturation at 94 °C for 3 min; 35 cycles of denaturation at 94 °C for 30 sec, annealing at temperatures listed in Table 5 for 30 sec, extension at 72 °C for 1 min/kb; and final extension at 72 °C for 10 min. Two PCR reactions for each sample were prepared, one was used to detect large deletions while the other was used for Surveyor^®^ mutation detection assay to detect small indels. PCR products were resolved in a 2% agarose gel and compared to wild type controls to detect large deletions. Samples with large deletions showed shorter DNA bands(s) (Figs 1Aa, 2Aa). Surveyor^®^ mutation detection assay was performed using Surveyor^®^ mutation detection kit for standard gel electrophoresis (Integrated DNA Technologies) according to the manufacturer instructions^63^. A negative control reaction was included in the assay by using DNA from full-sib channel catfish that were not injected with gRNA/Cas9 protein. Surveyor^®^-digested samples were electrophoresed for 50 minutes in a 2% agarose gel using 1X TBE and compared to wild type samples (Figs 1Ab, 2Ab).

### DNA sequencing

Since the PCR product was expected to be a mixture of both wild-type and mutated alleles, cloning of the PCR product before sequencing was necessary. For TICAM 1, PCR was performed on six mutant individuals that were previously identified with PCR or Surveyor^®^ assay using the primers T2F (forward) and T2R (reverse), then PCR products from these individuals were pooled before the cloning step. For RBL, primers RBL F (forward) and RBL R (reverse) were used to amplify a segment from six mutant individuals for sequencing. PCR products for RBL were individually cloned. Cloning was performed using TOPO^®^ TA cloning^®^ kit for sequencing following the manufacturer’s instructions (Invitrogen, Carlsbad, CA). For each gene, DNA from three wild type individuals that were full-siblings to the mutated individuals was amplified using the same primers, then pooled into one reaction and cloned as a wild type control for sequencing.

One Shot^®^ TOP10 Electrocomp™ *E. coli* (Invitrogen, Carlsbad, CA) were then transformed with the pCR™4-TOPO^®^ vector containing the PCR product following the manufacturer’s instructions with some modifications. Two microliters of the TOPO^®^ cloning reaction were added to 25 µl of competent cells, kept on ice for 30 minutes, heat shocked at 42 °C for 30 sec, then kept on ice for 2 minutes before adding 250 µl of the SOC medium (provided with the kit) and incubated at 37 °C for 1 h. Selection of transformed cells was achieved by plating 50 µl and 100 µl on LB agar plates containing 100 ppm ampicillin and overnight incubation at 37 °C. Single colonies were picked up and inoculated into 1.5 ml eppendorf tubes containing 1 ml LB medium with the same concentration of ampicillin and incubation conditions before sending the samples for sequencing. T2F primer was used for sequencing of TICAM 1 mutated samples while RBL F primer was used for RBL mutated samples. Mutation rates for individuals were calculated for replicates of different treatments for TICAM 1 and RBL in both dead embryos and 4-month-old fingerlings. Mutation rates were calculated as the number of mutated individuals detected by PCR or Surveyor^®^ assay in a replicate or treatment/total number of individuals in the same replicate or treatment * 100.

### Statistical analysis

The analyses of covariance (ANCOVA) for the effects of the embryo and fry density on embryo mortality, survival, hatching and fry survival were performed. No significant effects for differences in density were detected (*p* = 0.274). Embryo mortality % was calculated as the number of dead embryos in a group or treatment divided by the total number of embryos and multiplied by 100. Each embryo was assigned a value representing the time (dpf) of death. Survival % was similarly calculated as the number of alive embryos in a group or treatment divided by the total number of embryos and multiplied by 100. Survival curves for embryos in the five groups were compared using Kaplan-Meier test. Log Rank (Mantel-Cox) test was used for pairwise comparisons for embryo survival.

Hatching % was calculated as the total number of fry that have completed hatching/total number of embryos*100. Each fry was assigned a day representing the time (dpf) of hatch. Mean time to hatch for each group was compared using Kaplan-Meier test. Hatching percent for the five groups was compared using one-way ANOVA. All assumptions were satisfied.

For TICAM 1, when equality of variance for survival percent was not satisfied, Welch’s test was used and pairwise comparisons for survival percent among the five groups were conducted using Games-Howell test. To account for the time to death, survival curves of fry in the five groups were compared using Kaplan-Meier test and survival curves were plotted. Alive fry were assigned a value (18) representing the dpf in which the experiment on fry survival was terminated. Pairwise comparisons for survival time to death were performed using Log-Rank (Mantel-Cox) test. Histograms were generated using Microsoft Excel 2016. Plots of curves for embryo survival, time to hatch and fry survival were generated using GraphPad Prism version 7.00 for Windows (GraphPad Software, La Jolla, California, USA). Error bars in Figs 1–6 were calculated based on the results from the three replicates for each treatment and control groups. In each histogram and its corresponding survival plot, the embryos used in the analysis were the same (had the same age), where the age is provided in panels B, D and E of Figs 4–6 (days post fertilization, dpf). All statistical analyses were performed using SPSS 23.0 software (IBM Corporation, Armonk, NY). Statistical significance was set at *p* < 0.05, and all data were presented as the mean ± standard error (*SEM*).

## Electronic supplementary material

Supplementary S1

## Data Availability

All data generated or analyzed during this study are included in this published article (and its Supplementary Information file).
