# Supplementary material for: Effects of CRISPR/Cas9 dosage on TICAM1 and RBL gene mutation rate, embryonic development, hatchability and fry survival in channel catfish
Source: Sci Rep. 2018 Nov 7;8:16499. doi: 10.1038/s41598-018-34738-4 (PMC6220201; doi:10.1038/s41598-018-34738-4)
Supplement: Supplementary file 1 — Supplementary S1 [file 41598_2018_34738_MOESM1_ESM.pdf]

Supplementary Information

Effects of CRISPR/Cas9 dosage on TICAM1 and RBL gene mutation rate, embryonic development, hatchability and fry survival in channel catfish

Ahmed Elaswad[1,2,*], Karim Khalil[1,3,*], Zhi Ye[1], Zhanjiang Liu[1], Shikai Liu[1], Eric Peatman[1], Ramjie Odin[1], Khoi Vo[1], David Drescher[1], Kamal Gosh[1], Guyu Qin[1], William Bugg[1], Nathan Backenstose[1] & Rex Dunham[1]

[1] School of Fisheries, Aquaculture and Aquatic Sciences, Auburn University, Alabama 36849, USA.

[2] Department of Animal Wealth Development, Faculty of Veterinary Medicine, Suez Canal University, Ismailia 41522, Egypt.

[3] Anatomy and Embryology Department, Faculty of Veterinary Medicine, Cairo University, Giza 12211, Egypt.

* These authors contributed equally to this work.

*Correspondence and requests for materials should be addressed to Zhi Ye, School of Fisheries, Aquaculture and Aquatic Sciences, Auburn University, 203 Swingle Hall, Auburn, Alabama 36849, United States. **Tel**: +1 3348444786; **Fax**: +1 3348449208; zzy0008@tigermail.auburn.edu

Figure S1

A

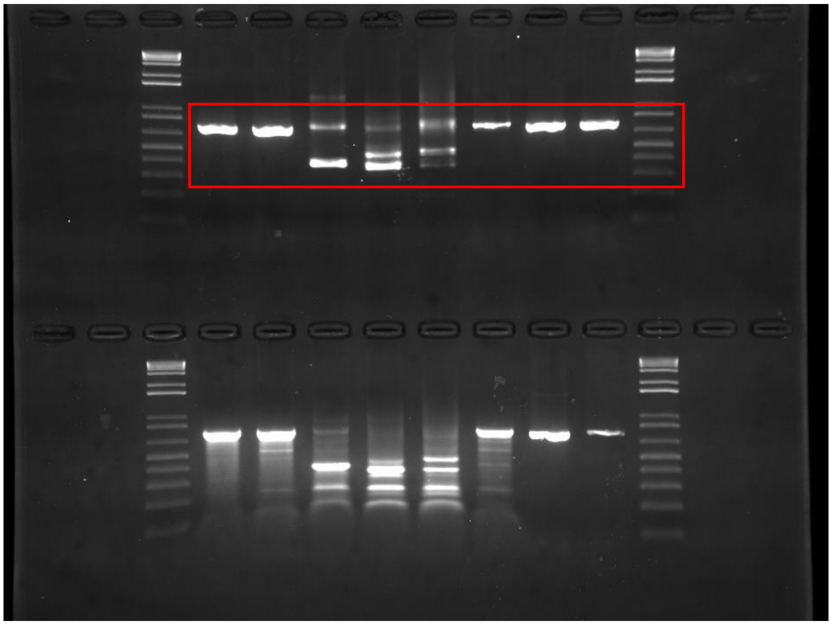

B

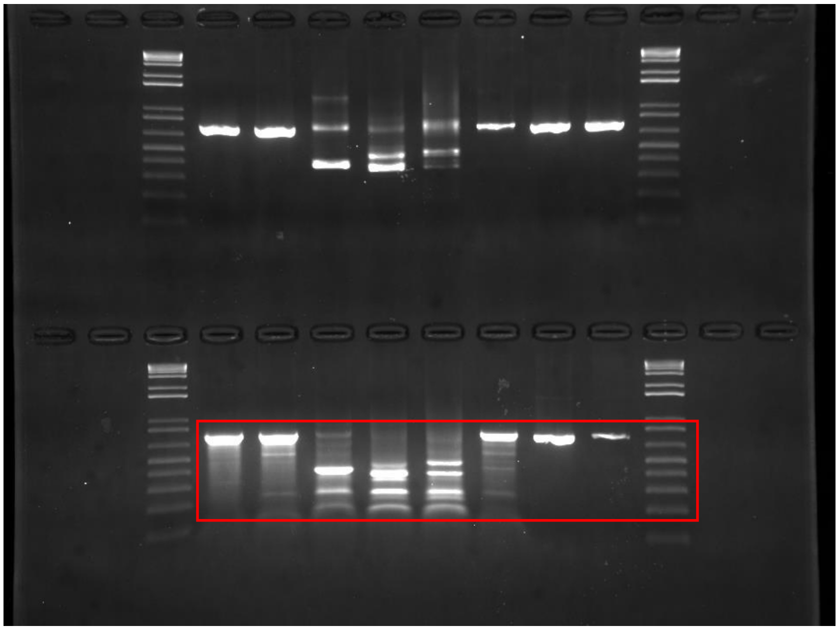

C

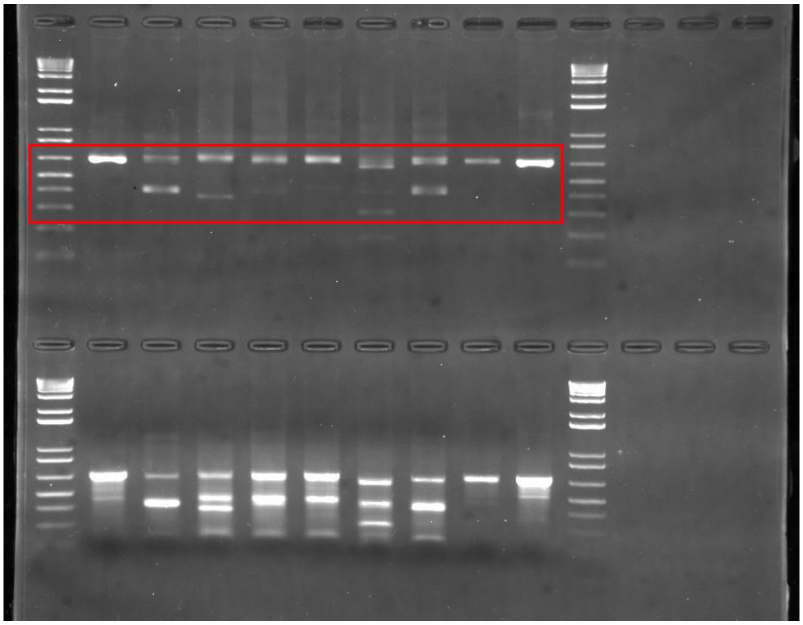

D

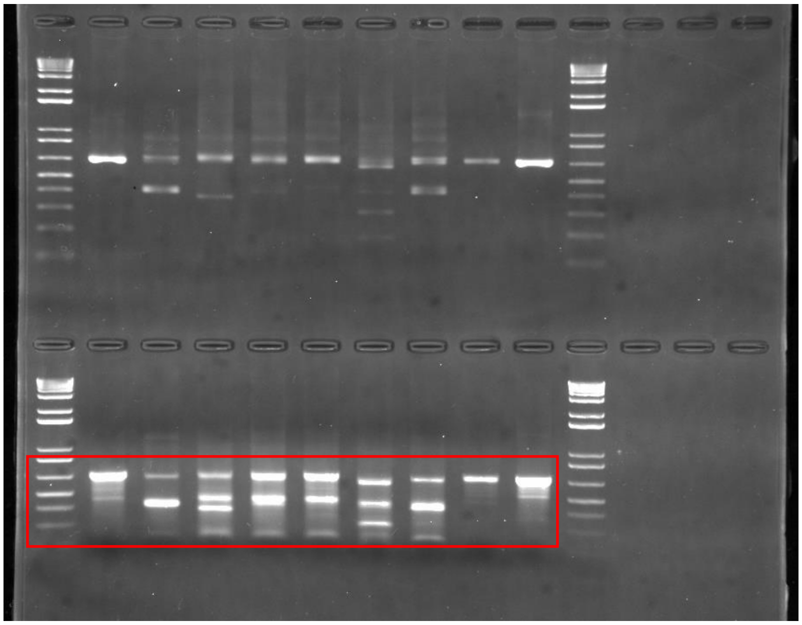

E

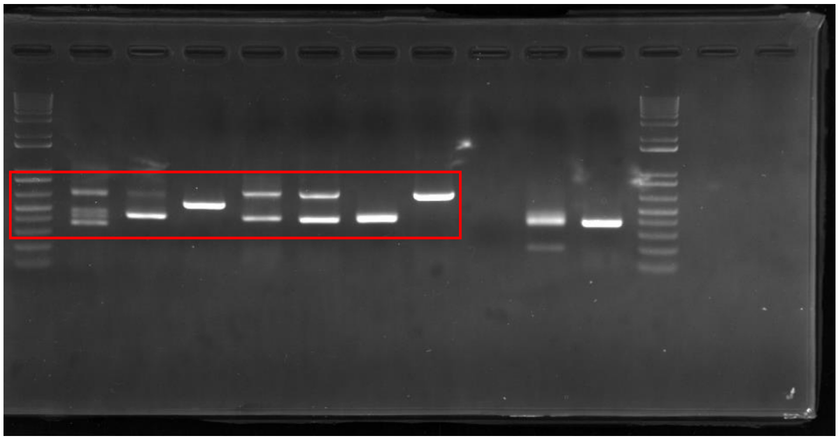

Supplementary Figure S1 original images of gels.

Results of PCR and Surveyor mutation detection in Fig. 1A (A, B) and Fig. 2A (C, D and E) are shown. Red box represents the cropped area.
